# Long-term warming and nitrogen fertilization affect C-, N- and P-acquiring hydrolase and oxidase activities in winter wheat monocropping soil

**DOI:** 10.1038/s41598-021-97231-5

**Published:** 2021-09-17

**Authors:** Chuang Zhang, Wenxu Dong, Kiril Manevski, Wenpei Hu, Arbindra Timilsina, Xiaoru Chen, Xinyuan Zhang, Chunsheng Hu

**Affiliations:** 1 Center for Agricultural Resources Research, Institute of Genetic and Developmental Biology, Chinese Academy of Science / Hebei Key Laboratory of Soil Ecology / Key Laboratory of Agricultural Water Resources, Chinese Academy of Sciences, Huaizhong Road 286, Shijiazhuang, 050022 China; 2 University of Chinese Academy of Sciences, Yuquan Road 19A, Beijing, 100049 China; 3 Department of Agroecology, Aarhus University, Blichers Allé 20, 8830 Tjele, Denmark; 4 Sino-Danish Center for Education and Research, Eastern Yanqihu Campus, Huaibeizhuang 380, Huairou, Beijing, 101200 China

**Keywords:** Hydrolases, Isoenzymes, Multienzyme complexes, Carbon cycle

## Abstract

The enzymatic activities and ratios are critical indicators for organic matter decomposition and provide potentially positive feedback to carbon (C) loss under global warming. For agricultural soils under climate change, the effect of long-term warming on the activities of oxidases and hydrolases targeting C, nitrogen (N) and phosphorus (P) and their ratios is unclear, as well as whether and to what extend the response is modulated by long-term fertilization. A 9-year field experiment in the North China Plain, including an untreated control, warming, N fertilization, and combined (WN) treatment plots, compared the factorial effect of warming and fertilization. Long-term warming interacted with fertilization to stimulate the highest activities of C, N, and P hydrolases. Activities of C and P hydrolase increased from 8 to 69% by N fertilization, 9 to 53% by warming, and 28 to 130% by WN treatment compared to control, whereas the activities of oxidase increased from 4 to 16% in the WN soils. Both the warming and the WN treatments significantly increased the enzymatic C:N ratio from 0.06 to 0.16 and the vector length from 0.04 to 0.12 compared to the control soil, indicating higher energy and resource limitation for the soil microorganisms. Compared to WN, the warming induced similar ratio of oxidase to C hydrolase, showing a comparable ability of different microbial communities to utilize lignin substrates. The relationship analyses showed mineralization of organic N to mediate the decomposition of lignin and enzyme ratio in the long-term warming soil, while N and P hydrolases cooperatively benefited to induce more oxidase productions in the soil subject to both warming and N fertilization. We conclude that coupled resource limitations induced microbial acclimation to long-term warming in the agricultural soils experiencing high N fertilizer inputs.

## Introduction

Global warming due to anthropogenic activities poses an ongoing concern for increased soil respiration and emission of carbon dioxide to the atmosphere^1^. Yet, scientific studies report thermal adaptation of the soil respiration to elevated temperature over the decades^2–5^. Microbial respiration is the essential constitute of soil respiration^4^ and the acclimation of the soil microorganisms to long-term warming is assumed to result from the depletion of soil water and labile carbon (C), which limits the microbial activities^6,7^ and shifts the community to utilize complex C compounds such as lignin^4,8,9^. Soil microbes meet their nutrient demands by producing a variety of functional enzymes to catalyze the mineralization of diverse organic matter. Soil enzymology provides insights into the decomposition of organic matter and therefore, the drivers of the soil C cycle^10^, promoting the scientific understanding of the mechanisms behind the microbial acclimation to global warming. The enzymology of agricultural soils is particularly interesting due to their high nitrogen (N) input from fertilization^11^, which might interactively with long-term warming affect the soil enzymes and consequently the soil C dynamics^11–13^.

Mineralization of soil organic matters involves both hydrolytic and oxidative reactions. Hydrolytic enzymes, i.e., hydrolases targeting soil decomposable organic carbon (SOC) include α-(*αGlu*) and β-1,4-glucosidase (*βGlu*), β-1,4-xylosidase (*βX*) and cellobiohydrolase (*CBH*), which decompose starch, cellulose, hemicellulose or xylose^14^. Whereas oxidases, such as peroxidase (*PER*) and polyphenol oxidase (*PPO*), decompose lignin with hydrogen (H) and oxygen (O) as electronic acceptor^15^. The dominant hydrolase in the soil capturing N and phosphorus (P) from chitin or phospholipid compounds in the soil are, respectively, β-1,4-*N*-acetylglucosaminidase (*NAG*) and alkaline phosphatase (*Phos*)^14^. The enzymatic ratio has been suggested as an efficient method to assess nutrient cycling and relative resource limitations for the soil microorganisms^16^. Macro-ecological studies show a balance distribution of enzyme C:N:P ratio, i.e., enzyme ratio of *βGlu:NAG:Phos* equal to 1:1:1^17,18^. However, it has also been reported that relatively high *Phos* activity in P deficient soils induces a deviation from the balance along climatic gradient^19,20^. Lin et al.^21^ found deviations up to 25% from the balance for 30-year fertilized soils. These studies imply that both biotic and abiotic factors such as changes in water contents, pH and chemical properties—substrate quality and nutrients availability, regulate the enzymatic activities and ratio^20,22^.

Recent meta-analyses show an increase in the oxidase activities (*PPO* and *PER*) due to long-term warming, without^10,23^ or with modest response of the hydrolase activities^13,24^, implying on the critical role of oxidase activities to meet microbial nutrient demands in the long-term warming soil. Mori^25^ argues that the calculation of the enzymatic ratio should consider the microbial utilization of the substrate, highlighting the use of oxidase activities in the calculation for soils under long-term warming. However, long-term warming increased both C-, N-, P-hydrolase and oxidase activities in a forest and a shrubland soil^26–28^. It therefore remains unclear how microorganism allocate oxidase or hydrolase to meet their nutrients demand in soils under long-term warming.

Nitrogen fertilization regulates the enzymes according to the microbial economic theory, that is, increasing the enzyme activities capturing relatively deficient nutrients^14^. For example, meta-analysis found that N fertilization increases the activities of C- and P-hydrolases^29,30^, without influencing those of N hydrolase^31–33^. However, indirect effects of N fertilization might drive enzyme activities, e.g., acidification caused by N fertilization restricting both C-, N- and P-hydrolase and oxidase activities^19^. Nitrogen fertilization under long-term warming can also increase drought and limitations for soil water due to increasing evapotranspiration, although microbial respiration is not necessarily affected^12^, which might be due to low response of the enzyme activities. Long-term warming depleted SOC^34^, and caused disproportional utilization of labile C^4^. Consequently, changing resource stoichiometry would cause shifts in enzymatic activities and ratio. Long-term warming interactively affected the activities of *Phos* in a grassland and meadow^35,36^, without stimulated activities of *βGlu* and *NAG* in a temperate grassland soil^35^. It is therefore important to clarify how N fertilization under long-term warming would regulate enzyme activities and ratio, particularly for semi-arid arable soil.

The main aim of the study was to investigate effect of decadal warming (W), fertilization (N) and their combination (WN), on the soil enzymology and ratios. Following the initial assumption of preferential utilization of labile C under long-term warming^4^, it was hypothesized that (1) long-term warming increase oxidase activities to meet microbial nutrient demands, reflecting microbial nutrient demands better than C hydrolase, and (2) N fertilization stimulate higher oxidase activities by alleviating soil N limitation, or decreased oxidase and C-, N- and P-acquiring hydrolase activities by exacerbating drought in the soil exposing to long-term warming.

## Results

### Soil physico-chemical properties

**Figure 1:**
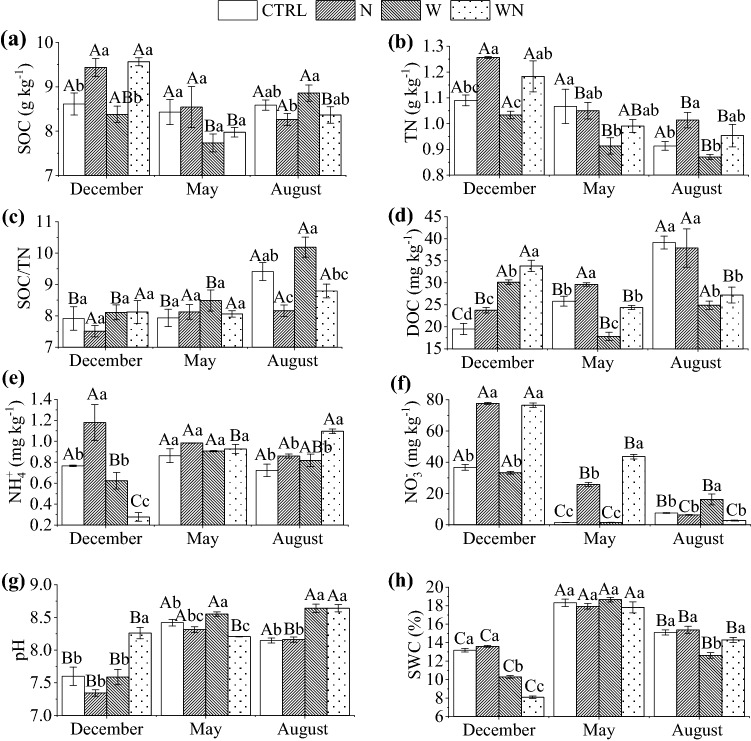
Effects of long-term warming (W), nitrogen fertilization (N) and their combination (WN) on soil physico-chemical properties. Small letters show significant differences between treatments in the same sampling time. Capital letters show significant differences between sampling times in the same treatment at 95% confidence level (n = 3, *p* < 0.05). The abbreviations are the same as in Table 3.

The treatment effects on the soil physico-chemical properties were more evident between than within sampling times (Fig. 1). The total nitrogen (TN) contents decreased 5 to 14%, on average, at all sampling times, in soils subject to warming compared to the CTRL and the effect was significant in May, resulting in a subsequent significant increase in the ratio SOC/TN in August. Relative to the CTRL, dissolved organic carbon (DOC) in two warming treatments (W and WN) was significantly higher (55–73%) in December, but significantly lower (3–31%) in May and August, reflecting intra-annual dynamics of this soil property to warming. Nitrate contents (NO_3_^−^) significantly increased in December and May due to the interacting effect of warming and N fertilization and increased by long-term warming in August, compared to CTRL, indicating accelerated N mineralization and nitrification, with concomitant decrease in soil water contents (SWC) for about 3–4%, whereas ammonium contents (NH_4_^+^) and soil pH were affected to a lesser degree by the treatments (Fig. 1).

### Soil enzyme activities and ratio

**Figure 2:**
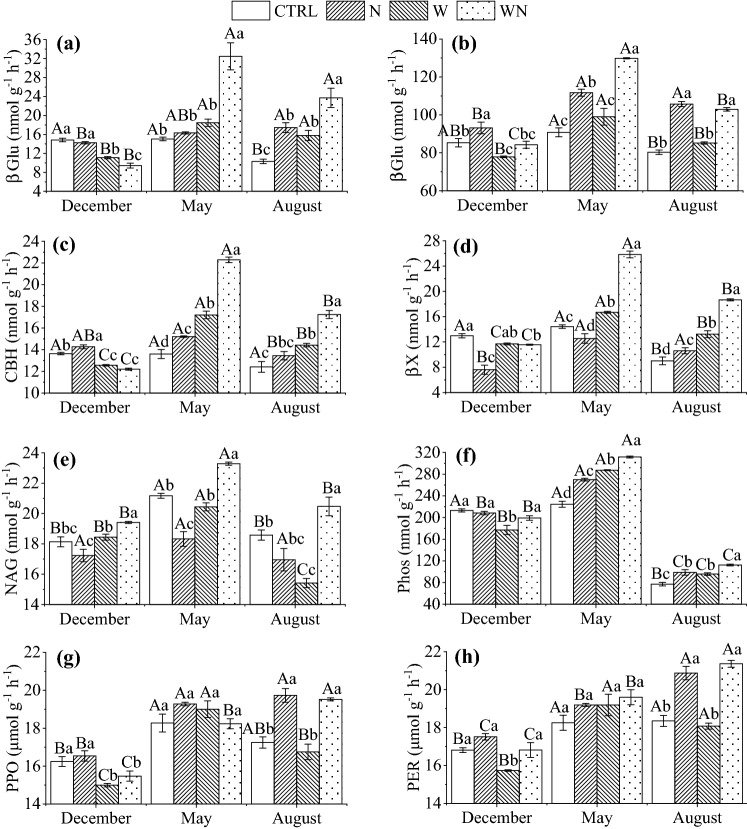
Effects of long-term warming (W), nitrogen fertilization (N) and their combination (WN) on soil C-, N-, and P-hydrolase and oxidase activities. *αGlu* and *βGlu* are, respectively, α- and β-1,4-glucosidase, *CBH* is cellobiohydrolase, *βX* is β-1,4-xylosidase, *NAG* is β-1,4-*N*-acetylglucosaminidase, *Phos* is alkaline phosphatase, *PER* and *PPO* are, respectively, per- and phenol oxidase.

Warming and N fertilization showed interactive effects on C-, N- and P-hydrolase activities, e.g., *αGlu* and *βGlu* in May or August, *CBH*, *βX* and *NAG* in the three sampling times, *Phos* in December and May (*p* < 0.05, Table 2). The activities of *αGlu*, *βGlu*, *CBH*, *βX*, and *Phos* were the highest in the WN soils in May and August, which were higher for 18–76%, 14–106% and 29–130% than, respectively, W, N and CTRL plots. In December, however, these enzyme activities in the two warming treatments (W and WN) were significantly lower for up to 36% compared to CTRL and N. In addition, the *NAG* activities in W and N decreased for 3–17% in May and August, which was significant for the N treatment in May and for the W treatment in August. In the WN plots, these enzymes activities significantly increased for 7–10% at all sampling times. The activity of *PPO* was more responsive to treatment between sampling time, whereas that of *PER* significantly increased from December to August (Fig. 2).

Between ‘seasons', the enzymatic ratios were consistently and often significantly higher in August compared to May and December, and this was particularly evident for the ratios involving C-oxidases (C_oxi_:*NAG* and C_oxi_:*Phos*) and *NAG*:*Phos* (Table 1). The interacting effect of N and W was more evident in December, whereas in May and August, when temperature and humidity are suitable for mineralization, sole treatment effects (N and W) were the largest and mostly evident for the C-hydrolases (Table 1). Within each season, December pointed mostly on long-term warming effects and only for the N-involving ratio, whereas May and August pointed on interactive effects between W and N on all ratios (Tables 1, 2, *p* < 0.01). Vector analysis showed about 0.13 higher vector length (L) and about 3.7 lower vector angle (A) in August than December and May, indicating higher C *vs*. nutrient and lower P *vs*. N limitation. Warming, N and WN increased A from 0.7 to 3.0, and increased L from 0.04 to 0.13 in May and August, while decreased A and L from 0.9 to 1.0 and from 0.04 to 0.06 in December.

**Table 1:** Enzymatic ratios and vector analysis in long-term warming (W), nitrogen fertilization (N) and combined treatments (WN).

Seasons	Treatments	C_hydro_:NAG	C_hydro_:Phos	C_oxi_:NAG	C_oxi_:Phos	NAG:Phos	Oxidase:C_hydro_	A (°)	L
December	CTRL	1.67 ± 0.00 Ab	0.90 ± 0.00 Ba	1.21 ± 0.01 Ab	0.65 ± 0.00 Ba	0.54 ± 0.00 Cb	0.72 ± 0.00 Ca	61.61 ± 0.12Aa	1.90 ± 0.00Bb
	N	1.71 ± 0.02 Ca	0.91 ± 0.00 Ca	1.24 ± 0.01 Ca	0.66 ± 0.00 Ba	0.53 ± 0.00 Bb	0.73 ± 0.00 Aa	61.94 ± 0.22Aa	1.94 ± 0.02Ba
	W	1.62 ± 0.01 Ac	0.91 ± 0.01 Ba	1.18 ± 0.01 Bc	0.66 ± 0.01 Ba	0.56 ± 0.01 Ba	0.72 ± 0.00 Ba	60.60 ± 0.30Bb	1.86 ± 0.00Cc
	WN	1.61 ± 0.01 Bc	0.90 ± 0.00 Ca	1.17 ± 0.01 Bc	0.66 ± 0.00 Ba	0.56 ± 0.00 Ba	0.73 ± 0.00 Aa	60.74 ± 0.12Ab	1.84 ± 0.01Cc
May	CTRL	1.60 ± 0.01 Bc	0.90 ± 0.00 Bb	1.18 ± 0.01 Bc	0.66 ± 0.00 Ba	0.56 ± 0.00 Ba	0.73 ± 0.00 Ba	60.58 ± 0.17Bd	1.84 ± 0.01Cc
	N	1.74 ± 0.02 Ba	0.90 ± 0.00 Bb	1.26 ± 0.01 Ba	0.65 ± 0.00 Bb	0.52 ± 0.00 Cd	0.72 ± 0.00 Ab	62.55 ± 0.19Aa	1.96 ± 0.02Ba
	W	1.66 ± 0.01 Ab	0.89 ± 0.01 Bc	1.21 ± 0.01 Bb	0.64 ± 0.00 Bc	0.53 ± 0.00 Bc	0.73 ± 0.00 Bb	61.94 ± 0.09Ab	1.88 ± 0.01Bb
	WN	1.70 ± 0.00 Ab	0.93 ± 0.00 Ba	1.15 ± 0.00 Bc	0.63 ± 0.00 Cd	0.55 ± 0.00 Bb	0.68 ± 0.00 Bc	61.27 ± 0.02Ac	1.94 ± 0.01Ba
August	CTRL	1.61 ± 0.00 Bc	1.09 ± 0.01 Aa	1.22 ± 0.00 Ab	0.82 ± 0.01 Aa	0.67 ± 0.00 Aa	0.76 ± 0.00 Aa	56.07 ± 0.14Cc	1.95 ± 0.01Ac
	N	1.77 ± 0.02 Aa	1.09 ± 0.01 Aa	1.31 ± 0.01 Aa	0.81 ± 0.01 Aab	0.62 ± 0.01 Ac	0.74 ± 0.00 Ab	58.36 ± 0.38Ba	2.07 ± 0.02Aa
	W	1.78 ± 0.01 Aa	1.07 ± 0.00 Aa	1.30 ± 0.00 Aa	0.78 ± 0.01 Ab	0.60 ± 0.01 Ac	0.73 ± 0.00 Ac	59.04 ± 0.25Ca	2.07 ± 0.01Aa
	WN	1.69 ± 0.01 Ab	1.08 ± 0.00 Aa	1.23 ± 0.01 Ab	0.79 ± 0.00 Ab	0.64 ± 0.01 Ab	0.73 ± 0.00 Ac	57.41 ± 0.25Bb	2.00 ± 0.01Ab

C_hydro_ is the sum of *αGlu*, *βGlu*, *CBH* and *βX*; C_oxi_ is the sum of *PPO* and *PER*. A is vector angle, L is the vector length. ln is log transformation. Small and capital letters show significant differences between treatments in the sampling time, and between sampling times in the same treatment, at 95% confidence level (n = 3, *p* < 0.05).

**Table 2:** Test of interactive effect between long-term warming (W) and nitrogen fertilization (N) on soil enzymatic activities and ratios in Decemeber, May and August.

Treatments	December			May			August		
	N	W	N*W	N	W	N*W	N	W	N*W
αGlu	**138.7**	**9.6**	ns	**43.1**	**26.3**	**18.2**	**21.1**	**34.8**	ns
βGlu	**14.9**	**11.2**	ns	**24.5**	**94.1**	ns	ns	**416.0**	**13.1**
CBH	**172.0**	ns	**16.6**	**309.8**	**121.1**	**33.0**	**60.9**	**26.8**	**5.8**
βX	**10.6**	**45.2**	**41.4**	**253.1**	**55.3**	**127.8**	**164.3**	**53.9**	**16.0**
NAG	**19.3**	ns	**11.0**	**52.5**	ns	**95.1**	ns	**10.5**	**39.9**
Phos	**19.0**	ns	**6.7**	**270.2**	**119.8**	**10.9**	**22.7**	**32.5**	ns
PPO	**24.4**	ns	ns	ns	ns	**6.4**	ns	**71.1**	ns
PER	**15.8**	**15.9**	ns	ns	ns	ns	ns	**127.2**	ns
C_hydro_:NAG	**44.2**	ns	**5.6**	ns	**57.8**	**18.7**	**8.3**	ns	**60.5**
C_hydro_:Phos	ns	ns	ns	ns	**28.2**	**37.5**	**6.8**	ns	ns
NAG:Phos	**28.5**	ns	ns	ns	**12.8**	**64.8**	**14.0**	ns	**64.3**
C_oxi_:NAG	**48.0**	ns	**6.3**	**17.3**	ns	**57.1**	ns	ns	**60.5**
C_oxi_:Phos	ns	ns	ns	**48.0**	**21.3**	ns	**20.1**	ns	ns

Values are F-ratios, bold indicates statistical significance at 95% confidence level (n = 18, *p* < 0.05).

### Relationships analyses

**Figure 3:**
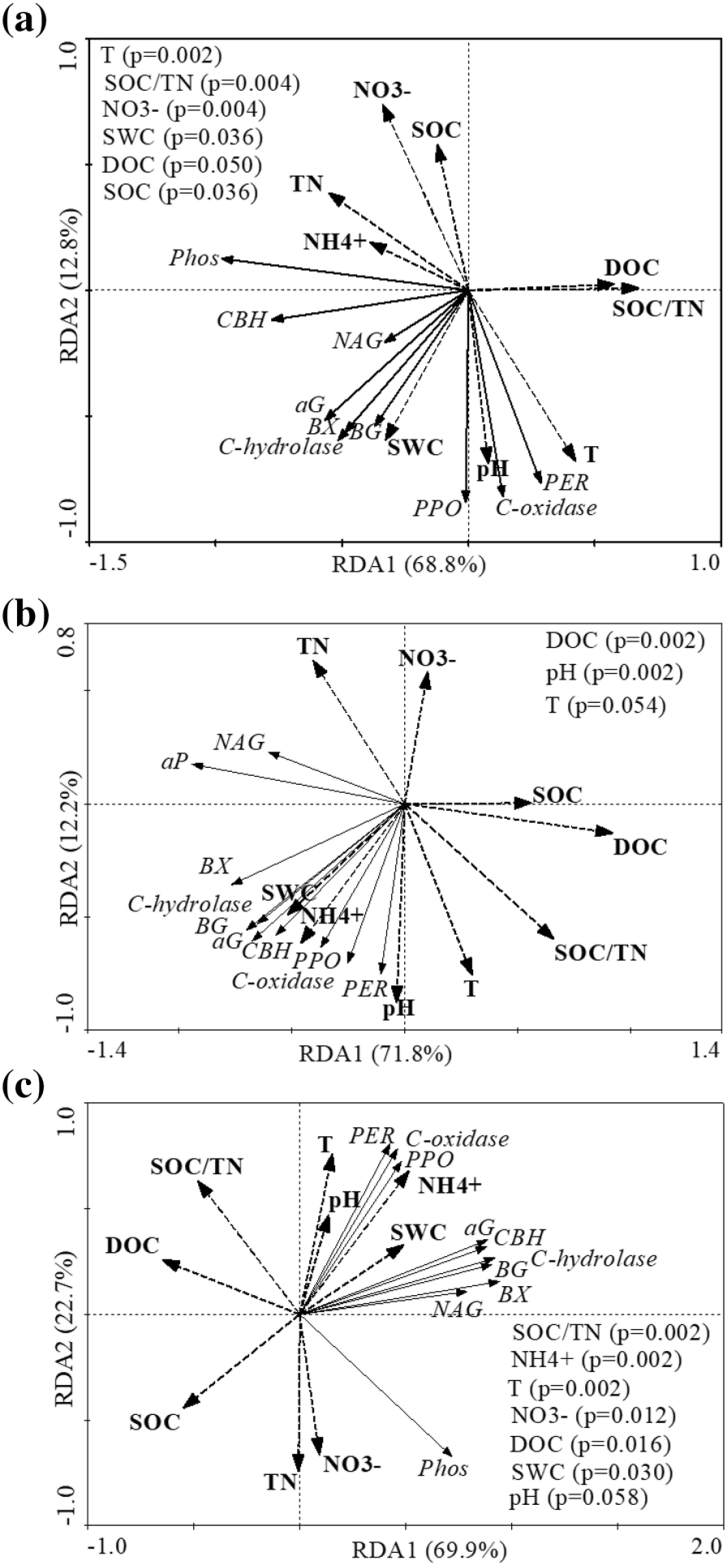
Redundancy analysis (RDA) between carbon (C)-, nitrogen (N)- and phosphorus (P)-hydrolase and oxidase activities and soil physico-chemical properties determined for long-term N fertilization (N, **a**), warming (W, **b**) and WN interaction (**c**). Listed variables of soil physico-chemical properties were significantly correlated with the first RDA axis (*p* < 0.05). The abbreviations are the same as in Table 3 and Fig. 2.

According to the redundancy (RDA) analysis, 69–72% of the variation in the enzymatic variables was contained in the first axis (RDA1) and the second axis explained 12–23% (Fig. 3). The RDA1 correlated with topsoil temperature (T) and DOC contents in the N, W and WN treatments (*p* ≤ 0.05). In W and WN plots, the activities of *PPO* and *PER* were negatively correlated with TN and NO_3_^−^ contents, in addition to their negative correlation with SOC in WN. Also, the activities of *αGlu*, *βGlu*, *CBH*, *βX* in W and WN plots were positively correlated with SWC and NH_4_^+^ and negatively correlated with SOC and DOC, while in W plots negative correlation was also seen for TN and NO_3_^−^ (Fig. 3).

In the W plots, the activities of *NAG* and *Phos* were positively correlated with TN and SWC and negatively correlated with soil T, SOC and DOC (Table 2), which points on a feedback of organic N mineralization on decomposition of SOC under long-term warming. However, in WN, *NAG* activities were negatively correlated with DOC and SOC, but *Phos* activities were positively correlated with TN and NO_3_^−^ contents, indicating a join positive effect of *NAG* and *Phos* on the decompositions of SOC. Therefore, in all warming plots (W and WN), increased *Phos* activities indiretcly factilicated organic N mineralization. In the N plots, the activities of *αGlu*, *βGlu*, *CBH*, *βX*, *NAG*, and *Phos* were all negatively correlated with DOC and SOC/TN, and positively correlated with SWC; the activities of *PPO* and *PER* were negatively correlated with SOC, TN, and NO_3_^−^ contents, and were positively correlated with pH, soil T and SWC contents (Fig. 3). This altogether indicates that the substrate contents and energy influence the enzymes activities in response to long-term N fertilization. The enzymatic ratios C_hydro_:*Phos*, C_oxi_:*Phos* and NAG:*Phos* were negatively correlated with TN and NO_3_^−^ contents (*p* < 0.01), and positively correlated with soil T (*p* < 0.05), expect for C_hydro_:*Phos* and NAG:*Phos* that positively correlated with DOC contents (*p* < 0.01, Table 3).

**Table 3:** Pearson analysis (n = 12) between enzymes ratio and soil physico-chemical properties across the three sampling times.

	lnC_hydro_:ln*NAG*	lnC_hydro_:ln*Phos*	lnC_oxi_:ln*NAG*	lnC_oxi_:ln*Pho*s	ln*NAG*:ln*Phos*
Soil T	ns	0.599**	0.358*	0.556**	0.506**
pH	ns	0.353*	ns	ns	ns
TN	ns	− 0.508**	ns	− 0.466**	− 0.473**
DOC	ns	0.463**	ns	ns	0.530**
NH_4_^+^	0.501**	ns	0.342*	ns	ns
NO_3_^−^	ns	− 0.481**	ns	-0.502**	− 0.471**

DOC is soil dissolved carbon, NH_4_^+^ and NO_3_^−^ are, respectively, soil ammonium and nitrate, SOC and TN are, respectively, soil organic carbon and total nitrogen, pH is soil acidity and SWC is soil water content. ns means no statistical significance and one (*) and two (**) asterisks denote statistical significance at, respectively, 95 (*p* < 0.05) and 99% (*p* < 0.01) confidence level.

## Discussion

Long-term warming and N fertilization had several distinct effects on the soil enzymology. Soils in the WN and the N plots had higher activities of oxidase compared to the CTRL, e.g., *PPO* and *PER* activities significantly increased in August (Fig. 2g,h). However, sole warming did not influence the oxidase activities (Fig. 2g,h), which disagreed with the first hypothesis that long-term warming increases the utilization of lignin substrate and consequently increases the activities of the oxidase in the soil^4, 5^. In support of the second hypothesis, long-term N fertilization interactively stimulated higher soils oxidase activities with long-term warming (*p* < 0.01) by mitigating soil N limitation (Table 2, 3). Moreover, the oxidase activities were negatively correlated with TN and NO_3_^−^ contents in W (Fig. 3b) and SOC, TN, and NO_3_^−^ in WN treatment (Fig. 3c), pointing on N contents in substrate as a limiting factor for oxidase under long-term warming, and therefore limiting the decomposition of lignin. Compared to the CTRL, the NO_3_^−^ contents in W plots indeed increased about 1.7 fold in August (Fig. 1), showing accelerated organic N mineralization and nitrification due to long-term warming^37^, which also feedback to the decomposition of lignin substrate^5,8,38^. Lower vector angle and higher length in August than December and May (Table 1) also showed increased microbial C and N demands relative to P in August in long-term warming plots. These findings implied that co-limitation of energy and N contents would be critical to SOC decomposition in a long-term warming soil^4,8^. Increased C and N demands of microorganisms might decelerate the C cycle in the future warming world^4,5^.

Although the soil in WN was characterized by the highest activities of oxidase (Fig. 2), the ability per unit of microbal biomass to utilize lignin substrates was comparable with that in warming plots, as implied by the similar ratio of oxidase to C-acquiring hydrolase activities (Table 1). The lack of response of oxidase activities to long-term warming could be attributed to the decrease in microbial biomass (see Fig. A1). However, increased oxidase activities did not cause loss of SOC in WN plots (Figs. 1a, 2g,h), partly because N fertilization stimulated accumulation of microbial residues in the soil exposing to long-term warming (unpublished data).

The C-hydrolase activities (*αGlu*, *βGlu*, *CBH* and *βX*) significantly increased in May and August in response to the treatments, with the largest increase observed in WN (Fig. 2), which fits the microbial economic theory for increased activity of enzymes acquiring relatively deficient C substances^14^. Decadal warming increases microbial C limitation^5,8^, as also supported by higher *C*_*hydro*_*:NAG* and *C*_*hydro*_*:Phos* ratio and higher vector length in warming and WN than CTRL (Table 1), consequently increasing the C hydrolase activities^26^. Nitrogen fertilization reduces microbial N demands and increased C and P demands^29,31^. In both W and WN plots, C-hydrolase activities were limited by energy and substrate contents, i.e., had negative correlation with SOC and DOC contents (Fig. 3b,c), pointing on energy and resource limitation promoting microbial thermal acclimation^5,8,38^. Considering the highest activities of C-, N- and P-acquiring hydrolase and oxidase in WN plots (Fig. 2), long-term N fertilization alleviate energy and resource limitation to microorganism by stimulating oxidase and hydrolase productions in the soils experiencing long-term warming^26^. Those were also evidenced by lower vector length and angle in WN compared to W treatments (Table 1).

In the growing season of winter wheat (May), the soil had lower enzymatic ratios of C:P, N:P and *Oxidase:C*_*hydro*_ compared to August (fallow season, Table 1), suggesting plant–microbe feedback to enzyme allocation, which rhizosphere exudates likely alleviated microbial C or energy limitation^39^. The W soil had significantly higher *C*_*hydro*_*:NAG* (August) and *C*_*oxi*_*:NAG* (May and August), *C*_*oxi*_*:Phos* (May) than the WN soil (Table 1). This result supported the first hypothesis that oxidase activities should be accounted to reflect intensive resource limitation^10,18,23,25^ due to the preferred utilization of labile C in the long-term warming^4,5,38^. However, comparing to the CTRL, the soil in W and WN had a lower ratio of Oxidase:C_hydro_ (May and August) and higher vector length (Table 1), suggesting microorganisms were more limited by energy than resource. It implies that microbial acclimation was more accounted by decreased microbial biomass^4,40^. The enzyme ratio of *C*_*hydro*_*:Phos*, *C*_*oxi*_*:Phos*, and *NAG:Phos* were negatively correlated with TN and NO_3_^−^ contents (*p* < 0.05, Table 3), suggesting that *Phos* activities can indirectly promote mineralization of soil organic N in warming and WN plots.

The activities of *NAG* and *Phos* significantly increased in the WN plots in May and August as compared to the CTRL plots (Fig. 2e,f) due to the interactive effects between N fertilization and warming (*p* < 0.05, Table 2). This result partly followed the microbial theory^14^ as N fertilization increased the activities of C- and P-acquiring hydrolase (Fig. 2). However, when combined with long-term warming, increase in *NAG* activities in WN plots was opposite to microbial economic theory. Compared to N or W treatments, lower vector angle (above 45°) and length in WN (Table 1) showed an increase in microbial N demands relative to C and P, which might explain increased NAG activities in WN. Before seeding, P fertilization was applied to all plots in order to minimize microbial P limitations in the soil^41^. In the WN plots, *NAG* activities negatively correlated with SOC and DOC contents, while *Phos* activities positively correlated with TN and NO_3_^−^ contents, but negatively correlated with DOC content (Fig. 3c). Consequently, increased activities of *NAG* and *Phos* were indirectly benefit to decomposition of soil organic matter in WN plots, meeting microbial C and energy demands^4,38^. *NAG* and *Phos* productions cooperatively promoted decompositions of SOC in the warming soil combined with N fertilization, and indirectly drove C cycle and might feedback to future global warming.

In the W treatment, *NAG* activities decreased in August and *Phos* activities increased in May and August (Fig. 2e). Moreover, the activities of *NAG* and *Phos* correlated negatively with SOC and DOC, but positively with TN (Fig. 3b), suggesting feedback of organic N mineralization to the decomposition of SOC under long-term warming^37,39^. This assumption is also supported by the increased NO_3_^−^ contents (Fig. 1) and *NAG* activities per unit of microbial biomass (Fig. A1) in August. Additionally, the activities of hydrolase and oxidase might have also been further limited by drought and alkalinity caused by long-term warming^14,42,43^. In W and WN, the activities of C, N, and P-acquiring hydrolase and oxidase were positively correlated with SWC and pH (Fig. 3b,c). However, this correlation might be insignificant because SWC decreased for rather small amount of 3% and soil pH increased for 0.4 units (Fig. 1g,h).

## Conclusion

Based on a near-decadal experiment, warming interactively with N fertilization affected C- and P-acquiring hydrolase activities, causing their highest activities. The activities of C-, N- and P-hydrolase in soils under combined WN were approximately 18–76%, 14–106% and 29–130% than in W, N and CTRL plots. Increased oxidase activities from 7 to 16% in WN caused similar oxidase to C hydrolase ratio in W and WN plots. Warming increased microbial C limitation (vector length) for about 0.04–0.13 in May and August. All together, the study showed energy rather than nutrient limitation, likely attributed to microbial acclimation to long-term warming. Nitrogen contents in substrate limited oxidase activities in long-term W plots, proven by a close relationship between total and available N and oxidase activities. In soils subject to both warming and N, cooperative limitation of C, N and P restricted oxidase activities, and positive correlation between P-acquiring hydrolase and total and available N and between N-acquiring hydrolase and organic C contents were found. Vector angle in WN was from 0.7 to 1.6 lower than in W, showing decreased P *vs.* N limitation. Enzymatic ratios of C:P and N:P negative correlated with total and available N (p < 0.05), indicating P-hydrolase indirectly benefit to organic N mineralization by meeting microbial energy demands. The estimations of C loss from fertilized agricultural soils under global warming should consider their interactive effects on hydrolase activities.

## Materials and methods

### Study site and experimental design

Long-term field experiment started in 2009 at Luancheng Agro-Ecological Station of the Chinese Academy of Sciences in Hebei Province, China (37° 53′ N, 114° 41′ E, 50 m above sea level). The site is characterized by semi-arid and monsoon-influenced climate, with mean annual temperature and precipitation of 12 °C and 460 mm, respectively. The soil is fluvo-aquic type formed by alluvial fan of limestone and is classified as Hapli-Ustic Inceptisols according to the US soil taxonomy^44^. The soil texture is sandy loam (54% sand, 34% silt, and 12% clay), with a bulk density of 1.3 g cm^−3^, pH of 8, organic matter content of 15 g kg^−1^ and total nitrogen (TN) content of 1 g kg^−1^ in the top 20 cm^41^.

The long-term warming and N fertilization experiment was a complete random block experimental design. Six plots (8 × 4 m) were randomly assigned as three untreated control (CTRL) and three fertilized with 240 kg^−1^ N ha^−1^ year^−1^ urea split to 50, 25 and 25% in November, March and May, respectively. Each plot was future divided in two sub-plots of 4 × 4 m and warming equipment was randomly assigned to one of the two subplots. Totally, 12 sub-plots (4 treatments × 3 replicates) included CTRL, warming (W), fertilization (N) and subject to both warming and fertilization (WN). The six sub-plots subject to W and WN involved heat simulated by infrared heaters of 2 m length and 2 cm width and rated power of 1000 W installed in triplets at about 2 m above the soil surface, yielding an effective radiation area of 2 × 2 m. The sub-plots without warming (CTRL and N) also contained the infrared heaters without connection to electrical power. Averagely, warming increased soil temperature for about 1.5 °C and caused 3% of decrease in soil water contents (SWC) compared to CTRL in the past 8 years^41,45^, which is similar to the estimated increase rate of global warming^1^.

Each year, the soils with residual stubble in all 12 plots were mechanically tilled to 40 cm depth and fertilized with Ca(H_2_PO_4_)_2_ of 65 kg P ha^−1^ year^−1^ in November before sowing. Irrigation was supplied with 60 mm of tap water in March and May, together with N fertilization. Winter wheat (selected from the local cultivars and complying with relevant institutional, national, and international guidelines and legislation) was cultivated from October to June, whereas from June to October soybean was grown the first 2 years^41^, which enhanced canopy growth caused by warming resulted in similar soil temperature to the CTRL plots, and was consequently replaced with fallow thereafter. We removed all other plants from the 12 experimental plots each year.

### Soil sampling and analysis

Topsoil sampling (0–10 cm) was conducted 9 years after the start of the experiment, i.e., in December 2017, May and August 2018, reflecting critical seasons of plant dormancy, growth and fallow. In each sub-plot, five cores of 5 cm diameter were collected and pooled into one sample, transported to laboratory in an insulated incubator and stored at 4 °C after sieving through a 2 mm mesh. The samples were used to measure soil physico-chemical properties and enzyme activities. The enzyme assays, ammonium (NH_4_^+^) and nitrate (NO_3_^−^) analyses were conducted within a week after sampling. Soil temperature was read by a kerosene thermometer injected 5 cm into the soil of each sampling points, with an average of five point in each plots. Soil water contents were determined gravimetrically after oven drying at 105 °C for 24 h, and the real pH was measured with a digital meter (10 g fresh soil:25 ml CO_2_-free distilled water). Dissolved organic C was measured by flow analyzer (Vario TOC select, Elementar, Germany) on the collected filtrates after mixing 10 g of fresh soil to 50 ml of CO_2_-free distilled water for 2 h. Filtrates from 50 ml solution of 1 mol l^−1^ KCl with 10 g fresh soil were used to measure NO_3_^−^ by spectrophotometer (UV-2450, Shimadzu, Japan) at 220 nm and 275 nm, and NH_4_^+^ on a flow analyzer (SMARTCHEM 140, Italy). Contents of TN and SOC were measured by an elemental analyzer (Vario MACRO cube, Elementar, Germany), after removing carbonate and hydro-carbonate from the air-dried soil with 1 mol l^−1^ of HCl.

### Enzyme assay

Using the 96-well assay microplate fluorescence method of Saiya-Cork et al^46^, four C hydrolases (Enzyme Commission number in brackets) were measured, i.e., *αGlu* (EC 3.2.1.20), *βGlu* (EC 3.2.1.21), *CBH* (EC 3.2.1.91) and *βX* (EC 3.2.1.37). Also, N-acquiring hydrolase (*NAG*, EC 3.2.1.20) and a P-acquiring hydrolase (*Phos*, EC 3.1.3.1) were measured. Briefly, Fresh soil of 1 g was homogenized in 150 ml of 50 mmol l^−1^ sodium acetate buffer at a pH of 8.5 (similar to the field pH), followed by addition of 200 μl of homogenate and 50 μl of 4-methylumbelliferone-(MUB) associated substrate (200 μmol l^−1^) to 96 black wells and incubation of the mixture in dark for 4 h at 20 °C. Simultaneously, control of sodium acetate buffer, soil and MUB-associated substrate were performed by the same procedure, in order to remove their confounding effect. Each sample was replicated eight times, the reaction was terminated using 10 μl of 1 mol l^−1^ NaOH for 1 min and the fluorescence values were read using a microplate fluorometer (Synergy H4, BioTek) at an excitation of 365 nm and emission of 450 nm. The hydrolase activities were calculated as nmol g^−1^ dry soil h^−1^.

Two oxidases were determined by spectrophotometry, i.e., *PPO* (EC 1.14.18.1) and *PER* (EC 1.11.1.7). The homogenate was prepared as described above and 600 μl of it with 150 μl of substrate were added to the 96 wells to measure the *PPO* activities. For the *PER* activity, additional 10 μl of 0.3% H_2_O_2_ were added to the mixture, which was then incubated in the dark at 20 °C for 5 h. After centrifuging at 3,000 rpm for 3 min, 250 μl of the supernatant were transferred to a 96-well transparent microplate and the absorbance values were red by microplate spectrophotometer (Synergy H4, BioTek) at 460 nm. The activities of *PPO* and *PER* were calculated as μmol g^−1^ dry soil h^−1^.

The C hydrolase activity (C_hydro_) was the sum activities of *αGlu*, *βGlu*, *CBH* and βX, whereas the oxidase activity (C_oxi_) was the sum activities of *PPO* and *PER*. The enzymatic ratio of C to N (C_hydro_:*NAG* and C_oxi_:*NAG*) and C to P (C_hydro_:*Phos* and C_oxi_:*Phos*), as well as ratio of N to P and of oxidase to C_hydro_ were calculated after log-transformation.

Microbial nutrient limitation was evaluated by vector analysis of C-, N- and P-acquiring hydrolase activities, and vector length (L) and angle (A, °) were calculated as follow:

$$\mathrm{L}=\sqrt{{\left({\text{LnC}}_{\text{hydro}}/{\text{LnNAG}}\right)}^{2}\text{+}{\left({\text{LnC}}_{\text{hydro}}/{\text{LnPhos}}\right)}^{2}}$$

$${\text{A}}(^\circ ) = {\text{DEGREES}}\left\{ {{\text{ATAN2 }}[({\text{ LnC}}_{{{\text{hydro}}}} /{\text{LnPhos}},{\text{ (LnC}}_{{{\text{hydro}}}} {\text{/LnNAG)}}]} \right\}$$

Vector length quantifies the C limitation relative to nutrient limitation. Vector angle is the arctangent of the line extending from the plot origin to point (C_hydro_:*Phos*, C_hydro_:*NAG*). Microbes are more limited by P than N when A > 45° and vice versa^16^.

### Statistical analyses

The data on enzyme activities and ratios, soil physical and chemical properties were first tested for normality distribution. The effect of W, N and their interaction on soil properties and enzyme activities and ratio was tested by two-factor analysis of variance (ANOVA) with Tukey pairwise comparisons (n = 6). Differences in treatments between and within sampling times were tested by one-way ANOVA, also with Tukey pairwise comparisons (n = 3). All tests were conducted at 95% confidence level (*p* < 0.05). The relationships between soil properties and enzyme activities, and enzyme ratio were investigated by redundancy analysis (RDA) using Canoco ver. 4.5 (n = 18), and Pearson tests using IBM SPSS ver. 19.0, respectively. Figures were prepared using Origin 2018 and values shown in the figures and tables are means ± standard errors.

## Supplementary Information

Supplementary Information 1.
