# Supplementary material for: Long-term warming and nitrogen fertilization affect C-, N- and P-acquiring hydrolase and oxidase activities in winter wheat monocropping soil
Source: Sci Rep. 2021 Sep 17;11:18542. doi: 10.1038/s41598-021-97231-5 (PMC8448830; doi:10.1038/s41598-021-97231-5)
Supplement: Supplementary file 1 — Supplementary Information 1. [file 41598_2021_97231_MOESM1_ESM.docx]

# Annex:

**Fig. A1** Effects of warming on soil C-hydrolase and oxidase activities and N- and P- acquiring enzyme activities normalized by total microbial biomass. The microbial biomass was measured by extracting phospholipid fatty acid.
